# Ruthenium^II^(*η*^6^-arene) Complexes of Thiourea Derivatives: Synthesis, Characterization and Urease Inhibition

**DOI:** 10.3390/molecules19068080

**Published:** 2014-06-16

**Authors:** Muhammad Hanif, Muhammad Azhar Hayat Nawaz, Maria V. Babak, Jamshed Iqbal, Alexander Roller, Bernhard K. Keppler, Christian G. Hartinger

**Affiliations:** 1 School of Chemical Sciences, University of Auckland, Private Bag 92019, Auckland 1142, New Zealand; 2 Department of Chemistry, COMSATS Institute of Information Technology, Abbottabad 22060, Pakistan; 3 Centre for Advanced Drug Research, COMSATS Institute of Information Technology, Abbottabad 22060, Pakistan; 4 Institute of Inorganic Chemistry, University of Vienna, Waehringer Str. 42, Vienna 1090, Austria

**Keywords:** thiourea, triphenylphosphine, urease inhibition, Ru(arene) complexes

## Abstract

Ru^II^(arene) complexes have emerged as a versatile class of compounds to design metallodrugs as potential treatment for a wide range of diseases including cancer and malaria. They feature modes of action that involve classic DNA binding like platinum anticancer drugs, may covalent binding to proteins, or multimodal biological activity. Herein, we report the synthesis and urease inhibition activity of Ru^II^(arene) complexes of the general formula [Ru^II^(*η*^6^-*p*-cymene)(L)Cl_2_] and [Ru^II^(*η*^6^-*p*-cymene)(PPh_3_)(L)Cl]PF_6_ with *S*-donor systems (L) based on heterocyclic thiourea derivatives. The compounds were characterized by ^1^H-, ^13^C{^1^H}- and ^31^P{^1^H}-NMR spectroscopy, as well as elemental analysis. The crystal structure of [chlorido(*η*^6^-*p*-cymene)(imidazolidine-2-thione)(triphenylphosphine)ruthenium(II)] hexafluorophosphate **11** was determined by X-ray diffraction analysis. A signal in the range 175–183 ppm in the ^13^C{^1^H}-NMR spectrum indicates the presence of a thione rather than a thiolate. This observation was also confirmed in the solid state by X-ray diffraction analysis of **11** which shows a C=S bond length of 1.720 Å. The compounds were tested for urease inhibitory activity and the thiourea-derived ligands exhibited moderate activity, whereas their corresponding Ru(arene) complexes were not active.

## 1. Introduction

Medicinal inorganic chemistry is a relatively new subject, but an emerging area of research in drug discovery that employs metals as pharmacophores or introduces structural features not achievable with organic structures. The choice of metal, oxidation state and ligand system provides an excellent platform for the design of metal coordination compounds with a wide range of biological applications. Nowadays many metallodrugs are routinely used for the treatment and diagnosis of a variety of diseases. These include gold compounds that are successfully applied as antiarthritic drugs, platinum chemotherapeutics as anticancer agents and gadolinium complexes as MRI contrast agents [1]. Since the discovery of the anticancer agent cisplatin, the design paradigm of metal-based anticancer compounds has largely focused on DNA-targeting metal complexes, resembling the mode of action of cisplatin and derivatives. However, drug development involving metal complexes has recently shifted from DNA targeting toward protein targeting drugs. In recent years, a variety of metal complexes acting as enzyme inhibitors were developed [2]. One strategy is centered around conjugation of organic inhibitors to a metal scaffold which often results in enhanced activity and selectivity compared to the free ligand [3]. A metal ion can either act as a spectator in a kinetically inert complex while its ligands interact with the active site of an enzyme, contributing solely to the overall structure of the inhibitor, or the metal ion can actively participate in the binding event to the enzyme. 

Group 8 metals such as ruthenium and osmium have attracted considerable attention for the design of specific enzyme inhibitors due to their relatively slower ligand exchange kinetics [4]. Moreover, the versatile synthetic chemistry of ruthenium offers flexibility to incorporate desirable properties into enzyme inhibitors. Ru and Os complexes with ligand structures resembling the shape of staurosporine were developed by Meggers *et al.* and they were shown to be excellent kinase inhibitors [3]. Dyson and co-workers developed biologically active ruthenium complexes based on the Ru^II^(arene) scaffold. Compounds with phenoxazin- or anthracene-modified ligands serve as multi-drug resistance modulators, while complexes containing ethacrynic acid act as inhibitors of glutathione-S-transferase, an enzyme involved in the detoxification of cells from cytotoxins [5]. A series of metal-based topoisomerase inhibitors with hydroxyflavone ligands which can be considered as multi-modal anticancer agents with both the organic ligand fragment and the metal scaffold contributing to the biological activity of the molecule was reported. Other examples of enzyme inhibitors are ferrocene and ruthenocene derivatives of sulfonamide which turned out to be potent inhibitors of carbonic anhydrase [6].

Most of the Ru(arene) complexes developed for medicinal applications contain either monodentate ligands comprising *P* and *N* donor atoms or bidentate ligands having *N,N-*, *N,O-* and *O*,*O*-donor sets. Ligand systems based on sulfur donor atoms have so far received little attention. Giannini *et al.* have recently reported dinuclear Ru(arene) complexes in which thiolato moieties coordinated to metal centers acted as a bridge between two ruthenium centers [7,8,9].

Urease inhibitors received considerable attention for the development of antiulcer agents, in particular, those with antibacterial activity against *Helicobacter pylori*. Urease (urea amidohydrolase; EC 3.5.1.5) is the specific nickel-containing heteropolymeric enzyme involved in the hydrolysis of urea to carbamate and ammonia [10]. Urease belongs to the superfamily of amidohydrolases and phosphotriestreases. Metal complexes of Cu(II) [11], Zn(II) [11] and Bi(III) [12] are believed to inhibit urease by binding to the sulfhydryl groups of cysteines and possibly nitrogen donor atoms of histidine as well as oxygen atoms of aspartic and glutamic acids in the urease active site [13,14].

In an attempt to design Ru(arene) complexes as urease inhibitors, we present here the synthesis, characterization and biological evaluation of a series of Ru^II^ complexes of the general formula [Ru^II^(*η*^6^-arene)(L)Cl_2_] or [Ru^II^(*η*^6^-arene)(PPh_3_)(L)Cl]PF_6_, where L represents heterocyclic derivatives of thiourea, which is a known urease inhibitor [15]. 

## 2. Results and Discussion

### 2.1. Synthesis and Characterization

**Scheme 1:**
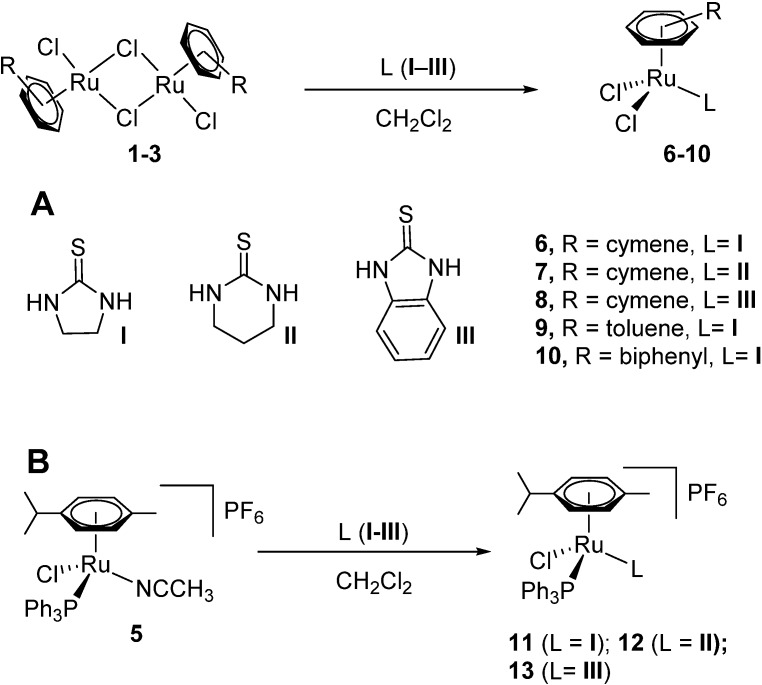
Synthesis of [Ru(arene)(L)Cl_2_] **6**–**10**; **B**) [Ru(arene)(L)(PPh_3_)Cl]PF_6_ **11**–**13**, where L = **I**, **II**, and **III** are imidazolidine-2-thione, 1,3-diazinane-2-thione and 1*H*-benzo[*d*]imidazole-2(3*H*)-thione, respectively.

The novel complexes **6**–**10** were obtained in good yields (63%–86%) by stirring two equivalents of the thiourea derivatives **I**–**III** with one equivalent of the respective ruthenium dimers **1**–**3** in dichloromethane or methanol for 2–4 h (Scheme 1). 

The structures of the complexes **6**–**10** were established by ^1^H- and ^13^C{^1^H}-NMR spectroscopy and the purity was confirmed by elemental analysis. In the ^1^H-NMR spectra, the signals originating from protons directly attached to the nitrogen atoms of the thiourea derivatives appeared at δ = 8–12 ppm, depending on the thiourea derivative and the solvent employed for the NMR measurement. In methanol-*d_4_* and DMSO-*d_6_*, additional species were observed after 24 h, probably due to ligand exchange with solvent molecules. Another reason could be the propensity of the sulfur donor atom to act as a bridging ligand. In the ^13^C{^1^H}-NMR spectra, the most significant signal was observed in the downfield region in the range of 170–184 ppm and was assigned to the C=S group of the thiourea ligands.

In order to extend the series, we also prepared the heteroleptic Ru^II^(arene) compounds **11**–**13** with triphenyl phosphine and a thiourea derivative as co-ligands. These compounds were prepared by replacing the labile acetonitrile ligand in [Ru^II^(*η*^6^-*p-*cymene)(acetonitrile)(PPh_3_)Cl]PF_6_ with the respective thiourea ligands **I**–**III** (Scheme 1), following a procedure reported earlier for carbohydrate-derived structurally related compounds [16].

**Figure 1:**
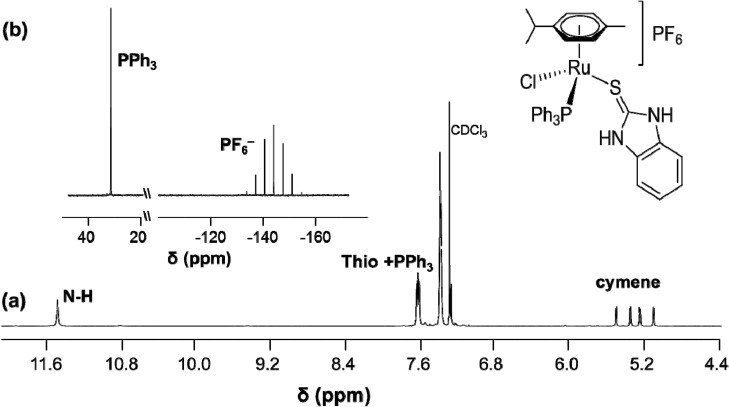
(**a**) The downfield region of the ^1^H-NMR spectrum of **13**. (**b**) ^31^P{^1^H}-NMR spectrum showing a singlet at 29.5 ppm (PPh_3_) and a septet at −144.2 ppm (PF_6_^−^).

Similarly to complexes **6**–**10**, the ^1^H-NMR spectra for **11**–**13** in aprotic solvents showed a characteristic peak in the δ = 8–12 ppm range for protons directly bound to nitrogen atoms (NH). The multiplets at δ = 7–8 ppm were assigned to the aromatic protons of the triphenylphosphine ligand. In **13**, the signals of the phenyl protons of the phosphine ligand overlapped with that of the aromatic protons of thiourea. The signal assigned to the *p*-cymene protons were observed as four doublets in the range of δ = 5–6 ppm. In the ^13^C{^1^H}-NMR spectra, the most diagnostic peak associated with the C=S group was found in the downfield region at δ = 181.3, 173.6 and 163.8 ppm for **11**, **12** and **13**, respectively, while the remaining PPh_3_ carbon signals appeared at δ = 128 to 134 ppm, and the aromatic carbons of the cymene ligand gave signals between 88 and 117 ppm. As an example, the NMR spectrum for **13** is shown in Figure 1. 

We previously reported that heteroleptic complexes featuring PPh_3_ and a chiral carbohydrate-derived phosphite were found to be present as a mixture of diastereomers as confirmed by ^31^P{^1^H}-NMR spectroscopy [16]. Due to the absence of a second stereocenter, **11**–**13** were present as enantiomers and the cymene-CH protons were inequivalent in the ^1^H-NMR spectrum, displaying four doublets. This is related to the slow epimerization of the chiral metal center and in line with the ^31^P{^1^H}-NMR spectrum revealing only a singlet signal at about 29 ppm for PPh_3_ and the septet signal at around −144 ppm for PF_6_ [17]. 

**Figure 2:**
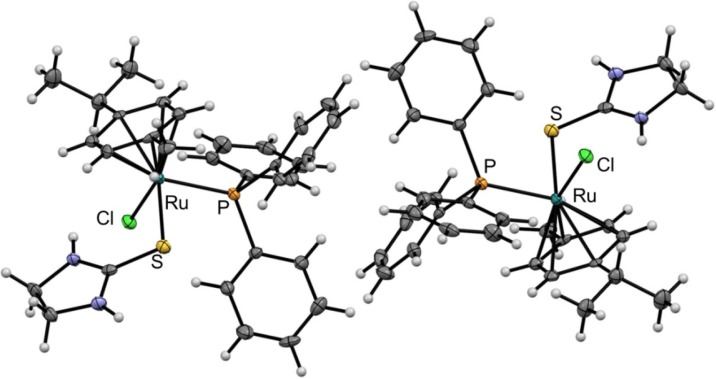
ORTEP diagram of the two enantiomers of **11** drawn at 50% probability level. The co-crystallized CH_2_Cl_2_ and PF_6_^−^ counter anions are not shown.

The molecular structure of **11** was established by single crystal X-ray diffraction analysis (see Table 1 for measurement parameters). Crystals of **11** suitable for X-ray diffraction analysis were grown by slow diffusion of diethyl ether into dichloromethane. The complex crystallizes in the triclinic *P*-1 space group and exhibits a typical piano-stool configuration with *p*-cymene forming the seat and the three co-ligands constituting the legs of the piano-stool structure. The crystal structures features two enantiomers in the cell (Figure 2). Selected bond lengths and bond angles are given in Table 2. The ruthenium–centroid_arene_ distance is 1.739 Å. The Ru–S bond length is 2.399 Å, while the C=S distance in thiourea is 1.720 Å indicating preference of a thione over a thiolate in the solid state. The Ru–Cl bond length is 2.415 Å, which is similar to that of structurally related compounds reported previously [18]. The Ru–P bond length is 2.369 Å and slightly elongated compared to the structurally related compound [Ru(*η^6^*-*p*-cymene)(PPh_3_)(PTA)Cl]^+^ (Ru–P 2.359 Å). 

**Table 1:** X-ray diffraction parameters for the measurement of single crystals of **11**.

Compound	11
CCDC N°	1001627
chemical formula	C_31_H_35_ClF_6_N_2_P_2_RuS·CH_2_Cl_2_
*M* (g mol^−1^)	865.06
temperature (K)	100(2)
crystal size (mm)	0.23 × 0.20 × 0.02
crystal color, habit	red, plate
crystal system	triclinic
space group	P-1
*a* (Ǻ)	9.7845(11)
*b* (Ǻ)	9.9124(8)
*c* (Ǻ)	18.1017(18)
*V* (Ǻ^3^)	1729.5(3)
*α* (deg)	81.773(5)
*β* (deg)	86.024(6)
*γ* (deg)	85.625(5)
*Z*	2
*D_c_* (g cm^−3^)	1.661
μ (mm^−1^)	0.898
F(000)	876.0
Θ range (deg)	2.23 to 25.34
*h* range	−11/11
*k* range	−11/10
*l* range	−21/21
No. unique refls.	6034
No. parameters	425
*R*_int_	0.1076
*R*_1_ (obs.)	0.0465
*wR*_2_ (all data)	0.1104
*S*	0.901

**Table 2:** Selected bond lengths (Å) and angles (°) of **11**.

Bond Lengths (Å)	
Ru–S	2.399(13)
Ru–P	2.369(12)
Ru–Cl	2.415(11)
Ru–centroid	1.739
**Bond Angles (°)**	
S–Ru–P	87.43(4)
S–Ru–Cl	90.11(4)
P–Ru–Cl	88.83(4)

### 2.2. In Vitro Urease Inhibition Assay

Thiourea derivatives **I**–**III **and their Ru(arene) complexes **6**–**13** were tested for their potential to inhibit jack bean urease as compared to thiourea as the positive control. The uncoordinated ligands exhibited moderate urease inhibition, while the ruthenium complexes were shown to be inactive (Table 3). Compound **II** was the most active in the tested series, with an IC_50_ value of 118 μM. This was, however, still significantly less active compared to thiourea, which was more than 5-times more active (IC_50_ = 22.1 μM ). The loss of activity of the ligands upon coordination to ruthenium may be attributed to their inability to bind to nickel at the active site of the enzyme. While in other cases coordination of a metal fragment to a biologically active enzyme inhibitor resulted in improved activity [3], in this study, the inhibitory activity of structural analogues of the natural substrate is diminished by such modification. 

**Table 3:** Inhibitory activity IC_50_ (μM) of thiourea derivatives and their Ru complexes against jack bean urease.

Compound	IC_50 _(μM)
**I**	232 ± 27
**II**	118 ± 13
**III**	˃600
**6**	314 ± 62
**7**	˃600
**8**	˃600
**9**	˃600
**10**	˃600
**11**	˃600
**12**	˃600
**13**	˃600
**Thiourea**	22.1 ± 1.4

## 3. Experimental Section

### 3.1. Materials and Methods

All reactions were carried out in dry solvents under inert atmospheres. All chemicals were obtained from commercial suppliers in analytical grade and used as received. The Ru complexes bis[dichlorido(*η*^6^-*p*-cymene)ruthenium(II)] (**1**), bis[dichlorido(*η*^6^-toluene)ruthenium(II)] (**2**), bis[dichlorido-(*η*^6^-biphenyl)ruthenium(II)] (**3**) [19], [dichlorido(*η*^6^-*p*-cymene)(triphenylphosphine)ruthenium(II)] (**4**) [20], and [Ru^II^(*η*^6^-*p*-cymene)(PPh_3_)(CH_3_CN)Cl]PF_6_ (**5**) [14], as well as the ligands imidazolidine-2-thione (**I**) [21], 1,3-diazinane-2-thione (**II**) [21], and 1*H*-benzo[d]imidazole-2(3H)-thione (**III**) [22] were synthesized according to literature procedures. ^1^H-, ^13^C{^1^H}- and ^31^P{^1^H}-NMR spectra were recorded at 25 °C on a Bruker FT NMR spectrometer Avance III 500 MHz at 500.10 (^1^H), 125.75 (^13^C{^1^H}) and 202.44 MHz (^31^P{^1^H}). Melting points were measured on a Büchi B-540 apparatus and are uncorrected. Elemental analysis data were determined by the Microanalytical Laboratory, Faculty of Chemistry, University of Vienna, on a Perkin–Elmer 2400 CHN Elemental Analyzer. X-ray diffraction measurements of single crystals were performed on a Bruker X8 APEX II CCD diffractometer at 100 K. The crystals were positioned at 35 mm from the detector and 1079 frames for 40 s over 1° were measured. The data was processed using the SAINT Plus software package [23]. Crystal data, data collection parameters, and structure refinement details are given in Table 1. The structures were solved by direct methods and refined by full-matrix least-squares techniques. Non-hydrogen atoms were refined with anisotropic displacement parameters. Hydrogen atoms were inserted at calculated positions and refined with a riding model. The following software programs and tables were used: SHELXS-97 [24], SHELXL-2013 [25], OLEX2 [26] and Mercury CSD 3.0 [27]. 

### 3.2. Urease Inhibition Assay

The urease inhibitory activity was measured according to a reported method [28] with slight modifications. The enzyme (5 U/mL; 10 μL) and test compound in buffer (10 μL) were sequentially added to 40 μL of buffer (pH 8.2) containing 100 mM urea, 1 mM EDTA, 0.01 M K_2_HPO_4_ and 0.01 M LiCl_2_. The mixture was pre-incubated for 30 min at 37 °C. The reaction was initiated by the addition of 40 μL of phenol reagent (1%, w/v phenol, 0.005%, w/v sodium nitroprusside) and 40 μL of alkali reagent (0.5%, w/v NaOH, 0.1% active chloride NaOCl) to each well. After 10 min of incubation at 37 °C, the absorbance was measured at 630 nm using a Microplate reader (Bio-Tek ELx 800^TM^, Instruments, Inc. USA). All the experiments were carried out with their respective controls in triplicate. Thiourea (0.1 mM well^−1^) was used as a positive control [29].

#### General procedure for the synthesis of [Ru^II^(*η*^6^-arene)(thiourea)C_l2_] compounds **6**–**10**

A solution of [Ru^II^(*η*^6^-arene)Cl_2_]_2_ (1 equivalent) and a heterocyclic thione (2 equivalents) was stirred in 20 mL dry dichloromethane (for **6**–**8**) or methanol (for **9**, **10**) for 3–4 h. The solution was concentrated under reduced pressure to a small amount (*ca.* 5 mL) and pentane or hexane was added to precipitate the product which was filtered, washed with pentane or hexane and dried *in vacuo*. All compounds were obtained as dark brown to red solids.

[Dichlorido(imidazolidine-2-thione)(*η*^6^*-p-*cymene)ruthenium(II)] (**6**). Compound **6** was synthesized by following the general procedure, using [Ru^II^(*η*^6^-*p*-cymene)Cl_2_]_2_ (**1**, 122 mg, 0.2 mmol) and imidazolidine-2-thione (**I**, 41 mg, 0.4 mmol). Yield: 109 mg (67%), m.p. 172–176 °C (decomp.), Elemental analysis (%) calcd. for C_13_H_20_N_2_SCl_2_Ru: C 38.24, H 4.94, N 6.86, S 7.85, found C 38.24, H 4.91, N 6.96, S 7.93. ^1^H-NMR (CDCl_3_): δ = 7.04 (s, 2H, NH), 5.40 (d, *J* = 6 Hz, 2 H, H_Ar_), 5.19 (d, *J* = 6 Hz, 2 H, H_Ar_), 3.66 (s, 4 H, CH_2_), 2.94–3.02 (m, 1 H, CH(CH_3_)_2_), 2.24 (s, 3 H, CH_3_), 1.32 (d, *J* = 7 Hz, 6 H, CH(CH_3_)_2_) ppm. ^13^C{^1^H}-NMR (CDCl_3_): δ = 184.1 (C=S), 105.8 (C_Ar_), 99.2 (C_Ar_), 86.0 (C_Ar_), 84.9 (C_Ar_), 83.7 (C_Ar_), 83.4 (C_Ar_), 45.5 (N-CH_2_), 31.3 (CH(CH_3_)_2_), 22.7 (CH(CH_3_)_2_), 19.7 (CH_3_) ppm.

[Dichlorido(1,3-diazinane-2-thione)(*η*^6^-*p*-cymene)ruthenium(II)] (**7**). Complex **7** was synthesized by following the general procedure, using [Ru^II^(*η*^6^-*p*-cymene)Cl_2_]_2_ (**1**, 122 mg, 0.2 mmol) and 1,3-diazinane-2-thione (**II**, 47 mg, 0.4 mmol). Yield: 106 mg (63%), m.p. 180–184 °C (decomp.). Elemental analysis (%) calcd. for C_14_H_22_N_2_SCl_2_Ru: C 39.81, H 5.25, N 6.63, S 7.59, found C 39.79, H 5.35, N 6.41, S 7.20. ^1^H-NMR (CDCl_3_): δ = 7.46 (s, 2H, NH), 5.36 (d, *J* = 6 Hz, 2 H, H_Ar_), 5.15 (d, *J* = 6 Hz, 2 H, H_Ar_), 3.24 (brs, 4 H, N-CH_2_), 2.94–3.03 (m, 1 H, CH(CH_3_)_2_), 2.22 (s, 3 H, CH_3_), 1.86 (brs, 2H, N-CH_2_-CH_2_), 1.31 (d, *J* = 7.0 Hz, 6 H, CH(CH_3_)_2_) ppm. ^13^C{^1^H}-NMR (CDCl_3_): δ = 172.6 (C=S), 103.3 (C_Ar_), 98.1 (C_Ar_), 87.2 (C_Ar_), 85.2 (C_Ar_), 83.4 (C_Ar_), 81.4 (C_Ar_), 40.2 (N-CH_2_), 30.4 (CH(CH_3_)_2_), 29.5 (N-CH_2_-CH_2_), 22.3 (CH(CH_3_)_2_), 18.2 (CH_3_) ppm.

[Dichlorido(1H-benzo[d]imidazole-2(3H)-thione)(*η*^6^-*p*-cymene)ruthenium(II)] (**8**). A mixture of [Ru^II^(*η*^6^-*p*-cymene)Cl_2_]_2_ (**1**, 184 mg, 0.3 mmol) and 1*H*-benzo[d]imidazole-2(3*H*)-thione (**III**, 90 mg, 0.6 mmol) was stirred in dichloromethane at room temperature for 3 h which resulted in the formation of a microcrystalline red precipitate. It was filtered, washed with dichloromethane (3 mL) and diethylether (3 × 5 mL), and dried *in vacuo*. Yield: 235 mg (86%), m.p. ˃200 °C (decomp.). Elemental analysis (%) calcd. for C_17_H_20_N_2_SCl_2_Ru·0.5H_2_O: C 43.87, H 4.55, N 6.02, S 6.89, found C 43.91, H 4.29, N 5.97, S 6.87. ^1^H-NMR (DMSO-*d_6_*): δ = 12.5 (s, 2H, NH), 7.11–7.15 (m, 4H, H_thio_), 5.81 (d, *J* = 6 Hz, 2 H, H_Ar_), 5.77 (d, *J* = 6 Hz, 2 H, H_Ar_), 2.80–2.89 (m, 1 H, CH(CH_3_)_2_), 2.09 (s, 3 H, CH_3_), 1.19 (d, *J* = 7 Hz, 6 H, CH(CH_3_)_2_) ppm. ^13^C{^1^H}-NMR (DMSO-*d_6_*): δ = 168.6 (C=S), 132.7 (C_thio_), 132.5 (C_thio_), 122.8 (C_thio_), 122.7 (C_thio_), 110.0 (C_Ar_), 106.8 (C_Ar_), 100.6 (C_Ar_), 86.8 (C_Ar_), 85.9 (C_Ar_), 30.4 (CH(CH_3_)_2_), 22.0 (CH(CH_3_)_2_), 18.2 (CH_3_) ppm.

[Dichlorido(imidazolidine-2-thione)(*η*^6^-toluene)ruthenium(II)] (**9**). Complex **9** was synthesized by following the general procedure, using [Ru^II^(*η*^6^-toluene)Cl_2_]_2_ (**2**, 158 mg, 0.3 mmol) and imidazolidine-2-thione (**I**, 61 mg, 0.6 mmol). Yield: 167 mg (76%), m.p. 168–172 °C (decomp.). Elemental analysis (%) calcd. for C_10_H_14_N_2_SCl_2_Ru: C 32.79, H 3.86, N 7.65, S 8.74, found C 32.77, H 3.81, N 7.50, S 8.83. ^1^H-NMR (DMSO-*d_6_*): δ = 7.98 (s, 2H, NH), 5.98–6.01 (m, 2 H, H_Ar_), 5.70–5.73 (m, 3 H, H_Ar_), 3.75 (s, 4 H, N-CH_2_), 3.50 (s, 3 H, CH_3_), ppm. ^13^C{^1^H}-NMR (DMSO-*d*_6_): δ = 183.0 (C=S), 106.0 (C_Ar_), 89.9 (C_Ar_), 85.3 (C_Ar_), 82.7 (C_Ar_), 44.5 (N-CH_2_), 19.0 (CH_3_) ppm.

[Dichlorido(imidazolidine-2-thione)(*η*^6^-biphenyl)ruthenium(II)] (**10**). Complex **10** was synthesized by following the general procedure, using [Ru^II^(*η*^6^-biphenyl)Cl_2_]_2_ (**3**, 163 mg, 0.25 mmol) and imidazolidine-2-thione (**I**, 51 mg, 0.5 mmol). Yield: 154 mg (72%), m.p. ˃200 °C (decomp.). Elemental analysis (%) calcd. for C_15_H_16_N_2_SCl_2_Ru: C 42.06, H 3.77, N 6.54, S 7.47, found C 41.72, H 3.84, N 6.65, S 7.37. ^1^H-NMR (DMSO-*d*_6_): δ = 8.50 (s, 2H, NH), 7.81–7.84 (m, 2H, H_bip_), 7.49–7.51 (m, 3H, H_bip_), 6.43–6.45 (m, 2H, H_bip_), 6.07–6.10 (m, 3H, H_bip_), 3.50 (s, 4 H, N-CH_2_) ppm. ^13^C{^1^H}-NMR (DMSO-*d*_6_): δ = 183.1 (C=S), 134.2 (C_bip_), 131.0 (C_bip_), 130.1 (C_bip_), 129.3 (C_bip_), 129.1 (C_bip_), 101.0 (C_bip_), 98.1 (C_bip_), 88.2 (C_bip_), 87.9 (C_bip_), 85.9 (C_bip_), 44.5 (N-CH_2_) ppm.

[Chlorido(*η*^6^-*p*-cymene)(imidazolidine-2-thione)(triphenylphosphine)ruthenium(II)] hexafluoro-*phosphate* (**11**). A mixture of imidazolidine-2-thione (**I**, 21 mg, 0.2 mmol) and [Ru^II^(*η*^6^-*p*-cymene)(PPh_3_)(CH_3_CN)Cl]PF_6_ (**5**, 144 mg, 0.2 mmol) in CH_2_Cl_2_ (20 mL) was stirred for 3 h at room temperature. The solvent was concentrated to a small amount (*ca.* 3 mL) and the product was precipitated by addition of pentane (*ca.* 20 mL). The orange yellow powder was filtered, washed with pentane (2 × 5 mL) and dried *in vacuo*. Yield: 142 mg (91%), m.p. 185–187 °C (decomp.). Elemental analysis (%) calcd. for C_31_H_35_ClF_6_N_2_P_2_RuS·CH_2_Cl_2_: C 44.43, H 4.31, N 3.24, S 3.71, found C 44.32, H 4.26, N 3.27, S 3.86. ^1^H-NMR (CD_3_OD): δ = 7.63–7.67 (m, 6 H, PPh_3_), 7.50–7.54 (m, 3 H, PPh_3_), 7.43–7.47 (m, 6H, PPh_3_), 5.66 (d, *J* = 6 Hz, 1 H, H-Ar), 5.54 (d, *J* = 6 Hz, 1 H, H-Ar), 5.44 (d, *J* = 6 Hz, 1 H, H-Ar), 5.40 (d, *J* = 6 Hz, 1 H, H-Ar), 4.86 (s, 4 H, N-CH_2_), 2.63–2.71 (m, 1 H, CH(CH_3_)_2_), 2.01 (s, 3 H, CH_3_), 1.15 (d, *J* = 7 Hz, 3H, CH(CH_3_)_2_), 1.10 (d, *J* = 7 Hz, 3H, CH(CH_3_)_2_) ppm. ^13^C{^1^H}-NMR (CD_3_OD): δ = 181.3 (C=S), 134.0 (C-PPh_3_), 133.1 (C-PPh_3_), 132.7 (C-PPh_3_), 130.6 (C-PPh_3_), 128.0 (C-PPh_3_), 114.0 (C-Ar), 100.5 (C-Ar), 93.5 (d, *J* = 4 Hz, C-Ar), 91.7 (d, *J* = 4 Hz, C-Ar), 90.8 (d, *J* = 4 Hz, C-Ar), 88.6 (d, *J* = 4 Hz, C-Ar), 45.9 (N-CH_2_), 30.3 (CH(CH_3_)_2_), 21.2 (CH(CH_3_)_2_), 20.4 (CH(CH_3_)_2_), 16.1 (CH_3_) ppm. ^31^P{^1^H}-NMR (CD_3_OD): δ = 28.8 (s, PPh_3_), −144.0 (sept, PF_6_) ppm.

[Chlorido(*η*^6^-*p*-cymene)(1,3-diazinane-2-thione)(triphenylphosphine)ruthenium(II)] hexafluoro-phosphate (**12**). A mixture of 1,3-diazinane-2-thione (**II**, 21 mg, 0.2 mmol) and [Ru^II^(*η*^6^-*p*-cymene)(PPh_3_)(CH_3_CN)Cl]PF_6_ (**5**, 144 mg, 0.2 mmol) in CH_2_Cl_2_ (20 mL) was stirred for 3 h at room temperature. The solvent was concentrated to a small amount (*ca.* 3 mL) and the product was precipitated by addition of pentane (*ca.* 20 mL). The orange yellow powder was filtered, washed with pentane (2 × 5 mL) and dried *in vacuo*. Yield: 132 mg (83%), m.p. 190–191 °C (decomp.). Elemental analysis (%) calcd. for C_32_H_37_ClF_6_N_2_P_2_RuS·CH_2_Cl_2_: C 45.09, H 4.47, N 3.19, S 4.31, found C 45.32, H 4.31, N 3.26, S 3.54. ^1^H-NMR (CDCl_3_): δ = 7.82 (s, 2H, NH), 7.55–7.59 (m, 6 H, PPh_3_), 7.47–7.51 (m, 3 H, PPh_3_), 7.41–7.44 (m, 6H, PPh_3_), 5.71 (d, *J* = 6 Hz, 1 H, H-Ar), 5.46 (d, *J* = 6 Hz, 1 H, H-Ar), 5.14 (d, *J* = 6 Hz, 1 H, H-Ar), 5.01 (d, *J* = 6 Hz, 1 H, H-Ar), 3.41 (brs, 4H, N-CH_2_), 2.69–2.77 (m, 1 H, CH(CH_3_)_2_), 2.00 (s, 3 H, CH_3_), 1.16 (brs, 2H, 2H, N-CH_2_-CH_2_), 1.18 (d, *J* = 7 Hz, 3H, CH(CH_3_)_2_), 1.13 (d, *J* = 7 Hz, 3H, CH(CH_3_)_2_) ppm. ^13^C{^1^H}-NMR (CDCl_3_): δ = 173.6 (C=S), 134.0 (C-PPh_3_), 132.8 (C-PPh_3_), 132.5 (C-PPh_3_), 130.9 (C-PPh_3_), 128.4 (C-PPh_3_), 128.1 (C-PPh_3_), 116.9 (C-Ar), 98.8 (C-Ar), 95.8 (d, *J* = 5 Hz, C-Ar), 89.7 (d, *J* = 5 Hz, C-Ar), 89.3 (d, *J* = 5 Hz, C-Ar), 88.7 (d, *J* = 5 Hz, C-Ar), 40.8 (N-CH_2_), 30.6 (CH(CH_3_)_2_), 29.7 (N-CH_2_-CH_2_), 22.5(CH(CH_3_)_2_), 21.1 (CH(CH_3_)_2_), 19.0 (CH_3_) ppm. ^31^P{^1^H}-NMR (CDCl_3_): δ = 29.9 (s, PPh_3_), −144.3 (septet, PF_6_) ppm.

[Chlorido(*η*^6^-*p*-cymene)(1H-benzo[d]imidazole-2(3H)-thione)(triphenylphosphine)ruthenium(II)] hexafluorophosphate (**13**). A mixture of 1*H*-benzo[*d*]imidazole-2(3*H*)-thione (**III**, 21 mg, 0.2 mmol) and [Ru^II^(*η*^6^-*p*-cymene)(PPh_3_)(CH_3_CN)Cl]PF_6_ (**5**, 144 mg, 0.2 mmol) in CH_2_Cl_2_ (20 mL) was stirred for 3 h at room temperature. The solvent was concentrated to a small amount (*ca.* 3 mL) and the product was precipitated by addition of pentane (*ca.* 20 mL). The orange yellow powder was filtered, washed with pentane (2 × 5 mL) and dried *in vacuo*. Yield: 146 mg (88%), m.p. ˃200 °C (decomp.). Elemental analysis (%) calcd. for C_35_H_35_ClF_6_N_2_P_2_RuS·0.5CH_2_Cl_2_: C 48.97, H 4.17, N 3.22, S 3.68, found C 49.02, H 4.38, N 3.29, S 3.74. ^1^H-NMR (CDCl_3_): δ = 11.46 (s, 2H, NH),7.58–7.62 (m, 6 H, PPh_3_), 7.34–7.37 (m, 10H, PPh_3_/Ar-thione), 7.24–7.27 (m, 3H, PPh_3_), 5.47 (d, *J* = 6 Hz, 1 H, H-Ar), 5.31 (d, *J* = 6 Hz, 1 H, H-Ar), 5.21 (d, *J* = 6 Hz, 1 H, H-Ar), 5.06 (d, *J* = 6 Hz, 1 H, H-Ar), 2.81–2.89 (m, 1 H, CH(CH_3_)_2_), 1.90 (s, 3 H, CH_3_), 1.18 (d, *J* = 7 Hz, 3H, CH(CH_3_)_2_), 1.16 (d, *J* = 7 Hz, 3H, CH(CH_3_)_2_) ppm. ^13^C{^1^H}-NMR (CDCl_3_): δ = 163.8 (C=S), 134.1 (C-PPh_3_), 132.0 (C-PPh_3_), 131.6 (C-Ar_thione_), 131.1 (C-Ar_thione_), 130.8 (C-PPh_3_), 128.4 (C-PPh_3_), 124.6 (C-Ar_thione_), 116.5 (C-Ar), 111.7 (C-Ar_thione_), 101.1 (C-Ar), 92.0 (d, *J* = 2 Hz, C-Ar), 91.3 (d, *J* = 4 Hz, C-Ar), 90.0 (d, *J* = 2 Hz, C-Ar), 89.0 (d, *J* = 4 Hz, C-Ar), 30.5 (CH(CH_3_)_2_), 22.5(CH(CH_3_)_2_), 21.4 (CH(CH_3_)_2_), 17.6 (CH_3_) ppm. ^31^P{^1^H}-NMR (CDCl_3_): δ = 29.5 (s, PPh_3_), −144.2 (sept, PF_6_) ppm.

## 4. Conclusions

Ru^II^(arene) complexes have attracted increasing interest for their applications in medicinal chemistry. We have synthesized organometallic Ru(arene) complexes with sulfur donor ligands as potential urease inhibitors. Compounds **6**–**10** were obtained by reacting two equivalents of thiourea derivatives with one equivalent of ruthenium arene precursors. In order to extend the series, the heteroleptic ruthenium compounds **11**–**13** were prepared by replacing labile acetonitrile in [Ru^II^(*η*^6^-*p-*cymene)(acetonitrile)(PPh_3_)Cl]PF_6_ (**5**) with thiourea derivatives. The analytical data of these compounds and the solid state structure of **11** confirmed the nature of the complexes. The compounds were tested for their inhibition of jack bean urease. However, the free thiourea derivatives turned out to be only moderately active as compared to thiourea used as a control. Their complexation to a ruthenium center resulted in a significant decrease of urease inhibitory activity. 
